# Atomic Layer Deposition-Assisted Construction of Binder-Free Ni@N-Doped Carbon Nanospheres Films as Advanced Host for Sulfur Cathode

**DOI:** 10.1007/s40820-019-0295-8

**Published:** 2019-08-02

**Authors:** Jun Liu, Aixiang Wei, Guoxiang Pan, Qinqin Xiong, Fang Chen, Shenghui Shen, Xinhui Xia

**Affiliations:** 1 Guangdong Provincial Key Laboratory of Functional Soft Condensed Matter, School of Materials and Energy, Guangdong University of Technology, Guangzhou, 510006 People’s Republic of China; 2 Department of Information Science, Xinhua College of Sun Yat-sen University, Guangzhou, 510520 People’s Republic of China; 3 Department of Materials Chemistry, Huzhou University, Huzhou, 313000 People’s Republic of China; 4 College of Materials and Environmental Engineering, Hangzhou Dianzi University, Hangzhou, 310018 People’s Republic of China; 5 Department of Chemistry, Zhejiang University, Hangzhou, 310027 People’s Republic of China; 6 State Key Laboratory of Silicon Materials, Key Laboratory of Advanced Materials and Applications for Batteries of Zhejiang Province, and Department of Materials Science and Engineering, Zhejiang University, Hangzhou, 310027 People’s Republic of China; 7 Key Laboratory of Advanced Energy Materials Chemistry (Ministry of Education), College of Chemistry, Nankai University, Tianjin, 300071 People’s Republic of China

**Keywords:** Atomic layer deposition, Nickel, N-doped carbon nanospheres, Sulfur cathode, Lithium–sulfur batteries

## Abstract

Rational design of hybrid carbon host with high electrical conductivity and strong adsorption toward soluble lithium polysulfides is the main challenge for achieving high-performance lithium–sulfur batteries (LSBs). Herein, novel binder-free Ni@N-doped carbon nanospheres (N-CNSs) films as sulfur host are firstly synthesized via a facile combined hydrothermal-atomic layer deposition method. The cross-linked multilayer N-CNSs films can effectively enhance the electrical conductivity of electrode and provide physical blocking “dams” toward the soluble long-chain polysulfides. Moreover, the doped N heteroatoms and superficial NiO layer on Ni layer can work synergistically to suppress the shuttle of lithium polysulfides by effective chemical interaction/adsorption. In virtue of the unique composite architecture and reinforced dual physical and chemical adsorption to the soluble polysulfides, the obtained Ni@N-CNSs/S electrode is demonstrated with enhanced rate performance (816 mAh g^−1^ at 2 C) and excellent long cycling life (87% after 200 cycles at 0.1 C), much better than N-CNSs/S electrode and other carbon/S counterparts. Our proposed design strategy offers a promising prospect for construction of advanced sulfur cathodes for applications in LSBs and other energy storage systems.

**Electronic supplementary material:**

The online version of this article (10.1007/s40820-019-0295-8) contains supplementary material, which is available to authorized users.

## Highlights

- Construct binder-free Ni@N-doped carbon nanospheres (Ni@N-CNSs) films were prepared and used as sulfur host.
- N-doped carbon and nickel layer work together to suppress shuttle of polysulfides.
- Ni@N-CNSs/S electrode shows enhanced rate performance and good cycling life.

**Electronic supplementary material:**

The online version of this article (10.1007/s40820-019-0295-8) contains supplementary material, which is available to authorized users.

## Graphical abstract

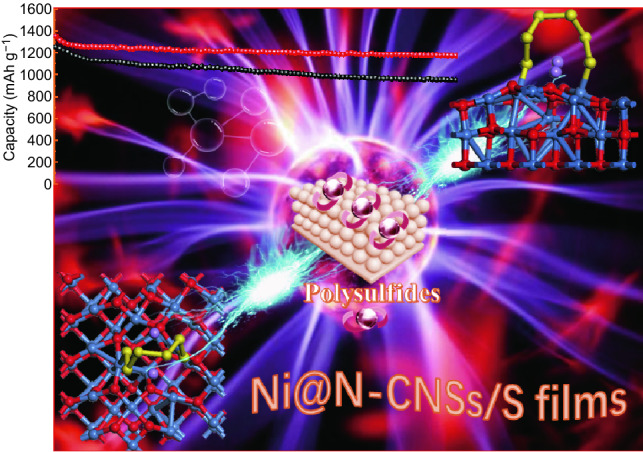

## Introduction

Over the past decades, great efforts have been made to develop advanced sulfur cathodes for lithium–sulfur batteries (LSBs) due to its high specific capacity (1675 mAh g^−1^), large theoretical energy density (2600 Wh kg^−1^) and low cost [1–4]. Despite promising prospect, the practical application of sulfur cathodes is still blocked by the following problems. (1) High-rate capability of sulfur cathode is not satisfactory on account of low electronic conductivity of active sulfur and final discharge product Li_2_S [5–7]. Their slow electron transfer not only decreases the reaction efficiency, but also leads to low utilization of active materials. (2) Cycling performance is poor due to the fact that the soluble long-chain lithium polysulfides intermediates show strong “shuttle effect” resulting in fast capacity decay [8]. (3) A volumetric expansion of ~ 80% happens to the sulfur cathode during full lithiation to Li_2_S, which is prone to cause structural damage and pulverization of active materials leading to inferior performance [9]. To address these problems, two main strategies including physical block and chemical bonding confinement are used to achieve high performance. One physical strategy is to accommodate sulfur into conductive matrixes/hosts [10–12], which not only act as physical barriers to retard the shuttle of lithium polysulfides, but also offer fast transfer paths for electrons. Additionally, another physical block way is using modified separators [13] or interlayers [14, 15] to suppress the shuttle of polysulfides. The latter two ways are usually used as the auxiliary means to restrain the loss of lithium polysulfides. Given all that, sulfur must be combined with advanced conductive hosts, which not only offer large space for accommodation of sulfur, but also possess high electrical conductivity, excellent physical block or chemical adsorption toward soluble lithium polysulfides. Typically, high-performance carbon host is still the first choice for sulfur by virtue of its lightweight, high conductivity, large storage space, cost-effectiveness, and easy modification on structure/composition.

Up to now, lots of carbon hosts (such as carbon nanofibers [16–19], reduced graphene oxides (rGO) [20, 21], carbon nanotubes [18], and carbon nano/micron spheres [22]) have been prepared and combined with sulfur to obtain enhanced performance. It is verified that the conductive carbon host can not only reinforce the electrical conductivity of the whole composite electrode, but also exhibit good suppressing effect toward the soluble lithium polysulfides arising from the physical block/adsorption via micro-, meso- or macropores of carbon [23]. For example, Lu et al. [24] reported 3D micron-porous graphene/sulfur composite cathode with enhanced capacity and good cycling life. In spite of enhanced performance to some extent, the shuttle effect of lithium polysulfides still cannot be completely stopped by the single physical block/adsorption of carbon hosts. In view of this situation, synergistic chemical adsorption strategy needs to be further introduced into carbon/S cathodes by adding polar chemical absorbents. There are two chemical adsorption ways to stabilize the soluble lithium polysulfides. One way is to introduce heteroatoms (e.g., N, P, and S) into carbon hosts forming heteroatom-doped carbon hosts [10, 25, 26]. The other way is to rationally combine polar metal (Ni, Co) [12] or compounds (metal oxides [27, 28], metal sulfides [29, 30], metal nitrides [20], and metal carbides [10], etc.) with carbon hosts forming polar hybrid hosts for sulfur. With the help of first-principle calculations, it has been demonstrated that the heteroatom-doped carbon and polar stoichiometric metal and compounds show higher binding energies (1.3–3.5 eV) with lithium polysulfides than that of the pure carbon hosts (0.5–1 eV) [27, 31]. It is well accepted that appropriate high binding energy can effectively suppress the shuttle of lithium polysulfides by chemical adsorption, resulting in higher capacity and better cycling life. Therefore, smart integration of heteroatom-doped carbon and polar chemical absorbents into integrated hybrid host is of great importance for achieving high-performance sulfur cathode.

Among the explored carbon hosts, carbon nanospheres (CNSs) have been widely studied as active hosts for sulfur. To date, different carbon nanospheres have been fabricated by different methods including hydrothermal synthesis with sacrificial silica/polystyrene templates, glucose decomposition [32, 33], thermal conversion via ZIF-8 template [34], and polyaniline-co-polypyrrole [35], combustion method [36], and sodium dodecyl sulfate-assisted self-assembly method [37]. However, the obtained carbon spheres are powder materials and need combining with additives and polymer binders to form working electrode. This process will increase inner resistance by introducing undesirable interfaces. Therefore, integrated binder-free carbon sphere films become attractive hosts due to binder-free characteristics and multiple blocking “dams” toward lithium polysulfides. To the best of our knowledge, there is no work on the synthesis of integrated N-doped carbon nanosphere (N-CNS) films as host for sulfur. Meanwhile, Ni metal is demonstrated having good chemical adsorption ability to lithium polysulfides. For example, Zhong et al. [12] embedded Ni nanoparticles into puffed rice carbon (PRC) forming PRC/Ni composites host for sulfur with enhanced rate capability. The implantation of Ni not only increases the electrical conductivity, but also synergistically suppresses the shuttling effect of polysulfides. However, their Ni nanoparticles prepared by immersion method show random sizes and cannot be controlled effectively. To overcome this problem, atomic layer deposition (ALD) emerges to produce Ni layer with high reproducibility and uniformity [38], as well as dead-space free. To date, there is no report on the rational combination between N-CNSs and ALD-Ni. Thus, it would be very interesting to explore the integrated Ni@N-CNSs films and their application as host for sulfur cathode.

In this work, we report novel binder-free Ni@N-CNSs films as host for sulfur by a powerful combined hydrothermal-ALD method. The thin ALD-Ni shell of ~ 10 nm is uniformly coated on the N-CNSs skeleton forming advanced host, which is highly compatible with sulfur forming integrated Ni@N-CNSs/S cathode. The Ni@N-CNSs films host not only exhibits high electrical conductivity and large storage room for sulfur, but also possesses synergistic chemical/physical adsorption toward lithium polysulfides. Due to the unique composite architecture, the Ni@N-CNSs/S cathode shows enhanced electrochemical performance with higher capacity, better cycling stability and excellent high-rate capability due to better physisorption and chemisorption abilities and higher conductivity. Our work demonstrates the synergistic effect between N-CNSs and Ni layer toward soluble lithium polysulfides.

## Experimental

### Preparation of N-Doped Carbon Nanospheres Films

The N-CNSs films were prepared by a modified hydrothermal method. The nickel foil coated with ZnO layer (~ 10 nm) was used as the substrate. The ZnO layer was prepared by atomic layer deposition (ALD, Picosun Oy) with Diethyl zinc (DEZ, 99.99%, Sigma-Aldrich) and H_2_O as the Zn and O precursors, respectively. Then, the above substrate was transferred into Teflon-lined autoclave liners, which contained hydrothermal reaction solution consisting of 0.25 M glucose and 0.1 M aniline in aqueous solution. The nickel foil substrate with ZnO layer was fixed and the autoclave was kept at 180 °C for 8 h. During the hydrothermal process, the sacrificial ZnO layer acted as an induced layer to make the glucose/aniline decompose and polymerize on the nickel foil to form carbon nanospheres. After rinse, the samples were annealed at 800 °C for 3 h in argon atmosphere to form N-CNSs films.

### Preparation of Ni@N-CNSs Composite Films

The Ni@N-CNSs composite films were prepared using a SUNALE R-200 ALD reactor (Picosun Oy) with Ni(Cp)_2_ and O_3_ (Ozone concentration ~ 10%, generated in a 500 sccm mixture of oxygen (99.99%) and nitrogen (99.998%) as sources for Ni and oxygen, respectively. The Ni(Cp)_2_ precursor was put in a stainless steel bottle kept at 165 °C, and the reaction chamber with N-CNSs films was kept at 300 °C and 14 kPa during the reaction. After ALD process, the samples were annealed at 400 °C for 2 h in mixture atmosphere (90% Ar + 10% H_2_) to form Ni@N-CNSs composite films.

### Preparation of Ni@N-CNSs/S Composite Cathode

The Ni@N-CNSs composite hosts and sulfur were put into the CO_2_ supercritical fluid infiltration reactor according to the weight ratio of 1.3:3. The pressure was 8.5 MPa and the reactor worked at 250 rpm and kept at 32 °C for 10 h. After release the bumped CO_2_ gas, the sample was transferred into Teflon-linked steel autoclave and kept at 155 °C for 12 h to obtain the final Ni@N-CNSs/S cathode. For comparison, the N-CNS/S sample was also prepared as the same condition. The load mass of S was about 2.5 mg cm^−2^ in the N-CNSs/S sample.

### Materials Characterization

Morphology and phase compositions of all samples were characterized by field emission scanning electron microscope (SEM, Hitachi S-4700), transmission electron microscope (TEM, FEI Tecnai G2 F20 at 200 kV), X-ray diffraction (XRD, Rigaku D/max 2550 PC (CuKα)), X-ray photoelectron spectroscopy (XPS, ESCALABMKLL spectrometer) and Raman spectra (Renishaw Raman microscope under 532 nm laser excitation). The content of sulfur was detected by thermogravimetric (TG) curves utilizing Netzsch STA 449C thermal analyzer.

### Electrochemical Characterization

The binder-free Ni@N-CNSs/S electrode was directly used as the cathode. 2025-type coin cells were applied to assemble test cells. N-CNSs/S electrode was prepared as the same procedure above. The electrolyte was 1 M bis(trifluoromethane) sulfonamide lithium salt (LiTFSI) in a mixed solvent of 1,3-dioxolane (DOL) and 1,2-dimethoxyethane (DME) with a volume ratio of 1:1, including 1 wt% LiNO_3_ as an electrolyte additive. The added electrolyte for each electrochemical cell was 20 μL mg^−1^. Lithium metal foil was used as the counter and reference electrode, and a polypropylene microporous film (Cellgard 2300) was used as the separator. 2025-type coin cells were assembled in a glovebox filled with Ar. The discharge/charge performances were tested on a Neware battery program-control test system in a potential range between 1.7 and 2.8 V at 25 °C. Cyclic voltammetry (CV) measurements were performed with a Princeton TMC 1000 electrochemical workstation (Princeton Applied Research, Co., LTD) in the potential range of 1.7–2.8 V (vs. Li/Li^+^) at a scan rate of 0.1 mV s^−1^. Electrochemical impedance spectroscopy (EIS) measurements were conducted in the frequency ranges from 100 kHz to 10 mHz by applying an AC signal of 5 mV on the Princeton electrochemical workstation. The specific capacity was calculated based on the mass of sulfur in the electrode.

### DFT Calculation

First-principle based density functional theory (DFT) was performed as implemented in the Vienna ab initio simulation package (VASP). The projector augmented wave (PAW) pseudopotentials and exchange correlation energy functional in generalized gradient approximation (GGA) with the Perdew–Burke–Ernzerhof (PBE) formulation was utilized. The kinetic energy cutoff was set as 500 eV. The k-point grids for carbon materials and NiO were 5 × 4 × 2. In this computation, the NiO (200) surface was modeled by a bottom fixed slab in a 2 × 2 supercell. The vacuum width was set to 15 Å for avoiding the interaction between adjacent slices due to the periodic boundary condition. The pure carbon, pyridinic-N were built by a bottom fixed slab in 3 × 2 supercell. The convergence tolerance for this atomic relaxation was set to 1.0 × 10^−4^ eV/atom for total energy and 0.01 eV Å^−1^ for force on each atom. The adsorption energies of optimized configurations were calculated by the equation of *E* = *E*_surf-Li2S6_ − *E*_Li2S6_ − *E*_surf_, where *E*_Li2S6_ was the energy of the free Li_2_S_6_ molecular, *E*_surf_ was the energy of the surface configuration of pure carbon, pyridinic-N, and NiO. *E*_surf-Li2S6_ was the total energy of the configuration with Li_2_S_6_ molecular on the corresponding surface.

## Results and Discussion

**Fig. 1:**
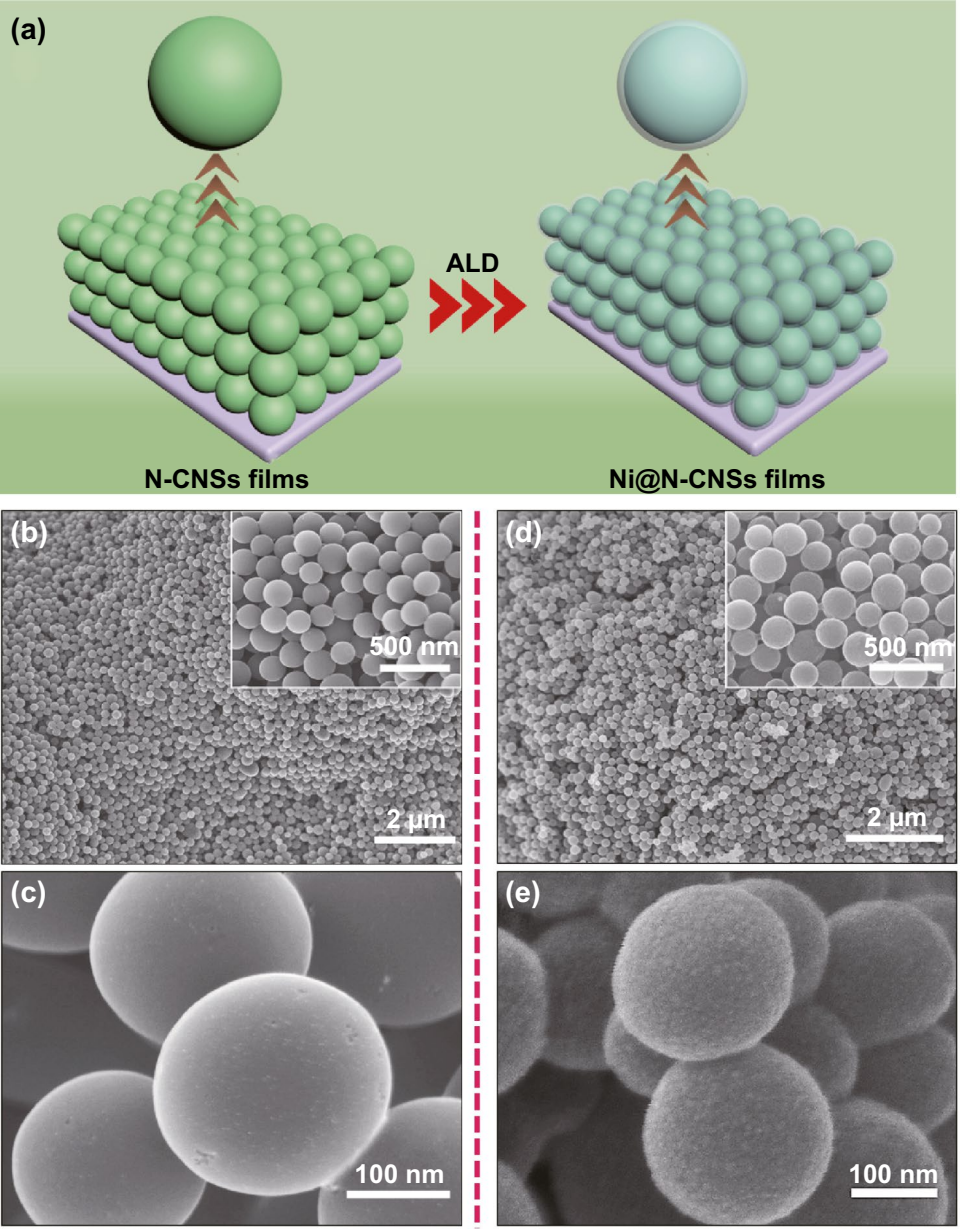
**a** Schematic fabrication process of Ni@N-CNSs films. SEM images of **b**, **c** N-CNSs and **d**, **e** Ni@N-CNSs films

**Fig. 2:**
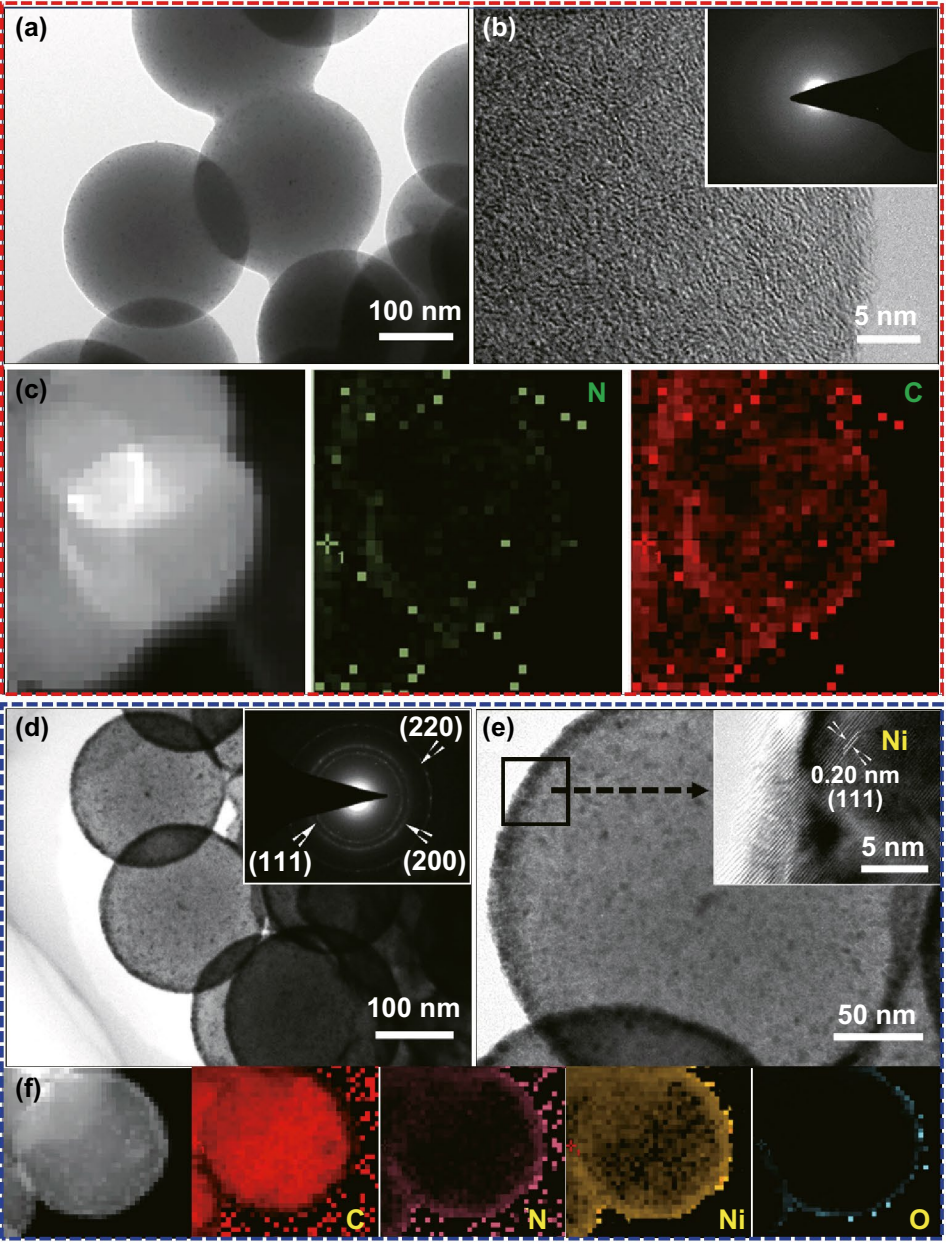
**a**, **b** TEM-HRTEM images and **c** EDS elemental mapping images of N-CNSs films. **d**, **e** TEM-HRTEM images and **f** EDS elemental mapping images of Ni@N-CNSs

Figure 1a illustrates the simplified synthetic process of Ni@N-CNSs films. Firstly, the N-CNSs films are fabricated via a modified hydrothermal method. As shown in SEM images (Fig. 1b, c), the N-CNSs films are composed of numerous cross-linked nitrogen-doped carbon nanospheres (N-CNSs) with average diameters of 250 nm. And interestingly, the as-synthesized N-CNSs are closely adhered to each other forming a continuous conductive network, which is favorable to accelerate the electron/ion transportation. Adsorption–desorption isothermal analysis shows that the surface area of N-CNSs films is about 206 m^2^ g^−1^ with a high porosity of about 80.5% (Fig. S1). ALD-synthesized nickel shell (~ 10 nm) is homogeneously deposited on the N-CNSs skeleton forming binder-free Ni@N-CNSs composite films (Fig. 1d, e). It is seen that the appearance of Ni@N-CNSs becomes rougher. The microstructures of samples at different stages are further investigated by TEM and HRTEM tests. The morphology of N-CNSs nanospheres is confirmed as shown in Fig. 2a. Additionally, according to the HRTEM image (Fig. 2b) and SAED pattern (inset in Fig. 2b), there is no obvious crystalline lattice fringe and diffraction rings detected, indicating the amorphous nature of N-CNSs. The doping of nitrogen element is evidenced by EDS elemental mapping images (Fig. 2c). After a facile ALD process, the N-CNSs are homogeneously decorated with Ni layer with a thickness of ~ 10 nm (Fig. 2d, e). The measured lattice space of Ni is ~ 0.20 nm (inset in Fig. 2e), corresponding to the (111) crystal plane of cubic Ni phase (JCPDS No. 04-0850). The polycrystalline nature of Ni shell is further verified by characteristic diffraction rings in SAED (inset in Fig. 2d). And the EDS elemental mapping images of Ni@N-CNSs films confirm that the Ni shell is uniformly covered on the surface of N-CNSs (Fig. 2f).

**Fig. 3:**
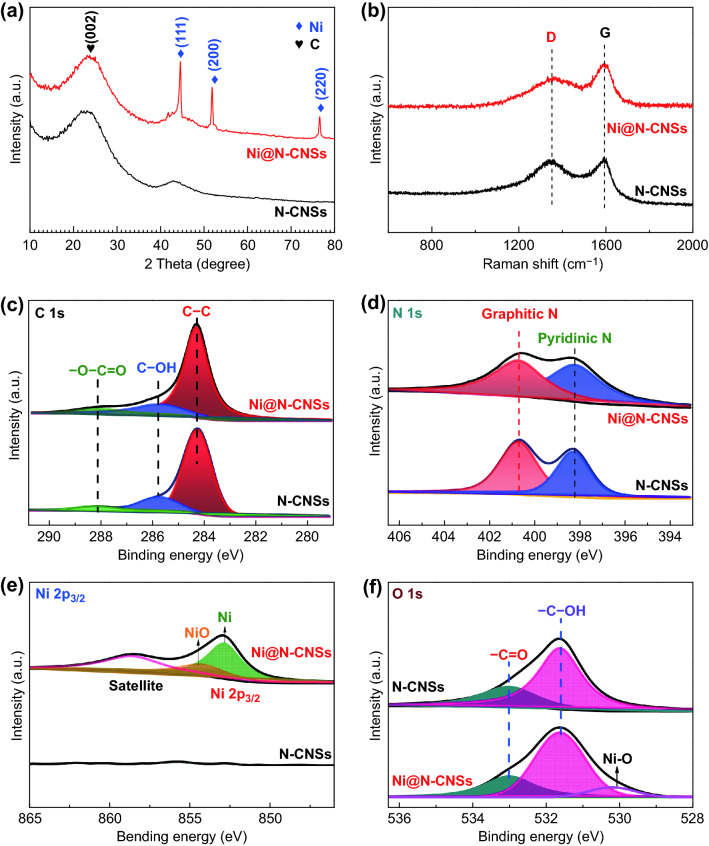
Phase and composition characterization. **a** XRD patterns; **b** Raman spectra; XPS test: **c** C 1*s* spectra; **d** N 1*s* spectra; **e** Ni 2*p* spectra; and **f** O 1*s* spectra of N-CNSs and Ni@N-CNSs electrodes

Detailed phase evolution and compositions of N-CNSs and Ni@N-CNSs are detected by XRD, Raman spectra, and XPS spectra (Fig. 3). The XRD pattern of N-CNSs (Fig. 3a) shows two broad diffraction peaks located at 26° and 43°, corresponding to the (002) and (101) crystal planes of carbon materials (JCPDS No. 75-1621). Apart from these peaks, the Ni@N-CNSs exhibits other three strong characteristic peaks, which can be indexed well with (111), (200), and (220) planes of cubic Ni phase (JCPDS No. 04-0850), confirming the successful deposition of Ni shell by ALD method. The above result is also supported by Raman results (Fig. 3b). It is noteworthy that the Ni metal does not show obvious Raman peaks. The Ni@N-CNSs shows larger *I*_G_/*I*_D_ ratio than pure N-CNSs, indicating its higher graphitization after the deposition of Ni shell and annealing process. The surface elemental composition and functional groups of samples are detected by XPS spectra (Fig. 3c–f). Both C 1*s* spectra (Fig. 3c) contain three characteristic peaks of –O–C=O (288.1 eV), C–OH (285.8 eV), and C–C (284.3 eV) bonds. The higher intensity of C–C peaks detected in Ni@N-CNSs verifies its higher graphitization, consistent with the Raman results above. For the N 1*s* spectra (Fig. 3d), two peaks located at 400.7 and 398.3 eV are detected in both samples, corresponding to graphitic *N* and pyridinic-*N*, respectively, which suggests that the *N* element is well maintained after the introduction of Ni shell. As for the Ni 2*p* spectra (Fig. 3e), it is noteworthy that characteristic peaks of Ni (852.9 eV) and NiO (854.5 eV) appear in Ni 2*p*_3/2_ spectra, demonstrating the existence of Ni and NiO arising from the surface oxidation of Ni by the air. For the O 1*s* spectra (Fig. 3f), two peaks of –C=O (533.0 eV) and –C–OH (531.6 eV) are noticed in both N-CNSs and Ni@N-CNSs, indicating that the surface of both samples contains –OH and –C=O groups. Meanwhile, a typical peak (530.1 eV) attributed to Ni–O bonding is detected in the Ni@N-CNSs due to the superficial oxidization layer of NiO on the Ni shell. All the results above demonstrate the successful synthesis of the Ni@N-CNSs array by a combined hydrothermal-ALD method.

**Fig. 4:**
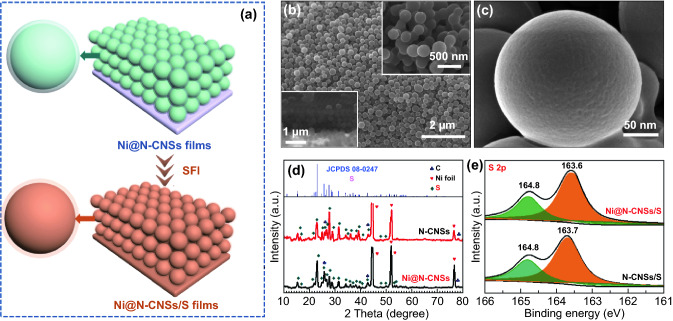
**a** Schematic fabrication process of Ni@N-CNSs/S electrode. **b, c** SEM images of Ni@N-CNSs/S electrode. **d** XRD patterns and **e** S 2*p* XPS spectra of N-CNSs/S and Ni@N-CNSs/S electrodes

**Fig. 5:**
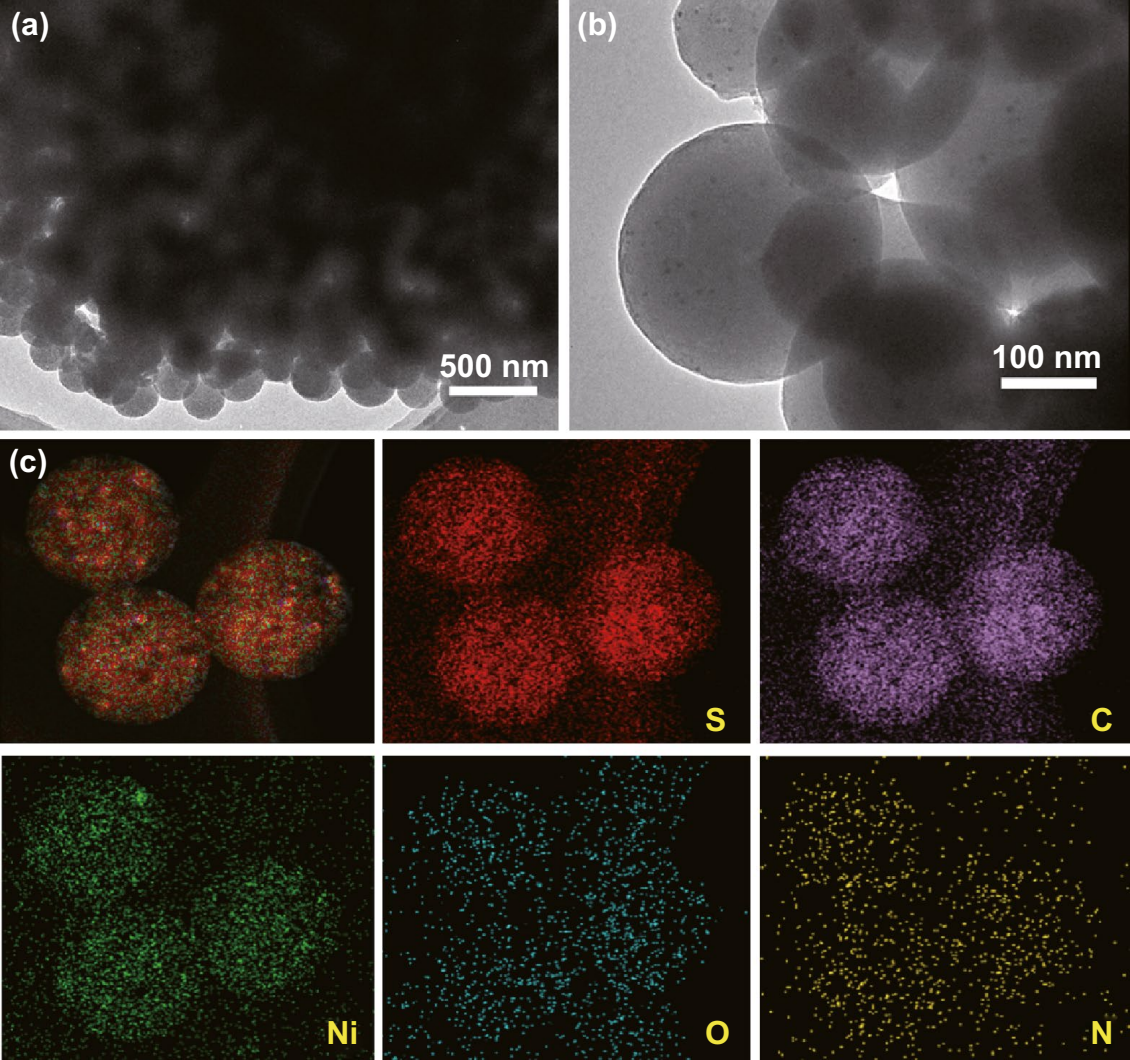
**a**, **b** TEM images and **c** EDS elemental mapping images of Ni@N-CNSs/S

To further explore the electrochemical application of N-CNSs and Ni@N-CNSs hosts for LSBs, sulfur is infiltrated into both hosts to obtain N-CNSs/S and Ni@N-CNSs/S via a supercritical fluid infiltration (SFI) method (Fig. 4a). Notice that sulfur is homogeneously accommodated into the carbon nanospheres and their diameter increases up to 280 nm with no aggregated sulfur particles (Fig. 4b, c). Similar morphology is observed for the N-CNSs/S sample (Fig. S2). The existence of sulfur in both samples can also be verified by XRD patterns (Fig. 4d). Apart from the peaks of Ni foil substrate and N-CNSs/Ni@N-CNSs hosts, a series of characteristic peaks of S are detected in both N-CNSs/S and Ni@N-CNSs/S, supported by XPS characteristic peaks [63.6 eV (2*p*_1/2_) and 164.8 eV (2*p*_2/3_)] of sulfur in the S 2*p* spectra (Fig. 4e). Additionally, TEM images of Ni@N-CNSs/S and N-CNSs/S (Figs. 5a, b and S3a) also demonstrate the good accommodation of sulfur in the Ni@N-CNSs and N-CNSs hosts. For Ni@N-CNSs/S, the Ni shell are homogeneously covered up due to the introduction of sulfur and the surface becomes smoother. EDS elemental mapping images of Ni@N-CNSs/S (Fig. 5c) also confirm the successful preparation of Ni@N-CNSs host. The element O is due to the presence of –OH and –C=O groups at the surface N-CNSs and superficial NiO at the surface of Ni layer, which is consistent with the XPS results above. From the TGA results (Fig. S3b), we can calculate that the contents of sulfur in N-CNSs/S and Ni@N-CNSs/S are 66.7 and 68.9 wt%, respectively.

**Fig. 6:**
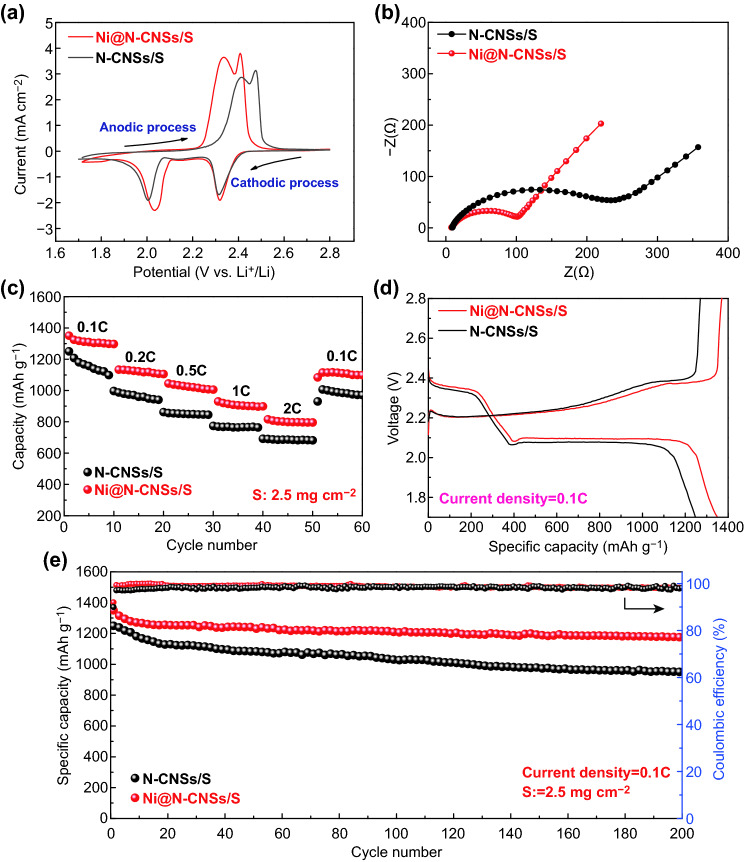
Electrochemical characterization of N-CNSs/S and Ni@N-CNSs/S electrodes. **a** CV curves at a scan rate of 0.1 mV s^−1^ at the second cycle. **b** Nyquist plots. **c** Rate capability with a sulfur loading of 2.5 mg cm^−2^. **d** Discharge/charge profiles at 0.1 C. **e** Cycling performance and coulombic efficiency at 0.1 C

The electrochemical performances of N-CNSs/S and Ni@N-CNSs/S electrodes are tested via cyclic voltammetry (CV) and galvanostatic charge/discharge measurements in a voltage range of 1.7–2.8 V at 25 °C. The overall electrochemical results are shown in Fig. 6. Figure 6a presents the 2nd CV curves of N-CNSs/S and Ni@N-CNSs/S electrodes at a scan rate of 0.1 mV s^−1^. Both electrodes exhibit two pairs of redox reaction peaks, which correspond to the conversion reactions of sulfur/long-chain polysulfides [S_8_/Li_2_S_*x*_ (*x* = 4–8)] and short-chain polysulfides/Li_2_S [Li_2_S_*x*_ (*x* < 4)/Li_2_S]. Obviously, the Ni@N-CNSs/S electrode exhibits higher peak density and smaller voltage separation with narrower polarization, indicating its higher reversibility and faster reaction kinetics during the charging/discharging processes. From EIS analysis (Fig. 6b), the charge transfer resistance of Ni@N-CNSs/S electrode is approximately 102 Ω, much smaller than that of N-CNSs/S electrode (220 Ω), indicating its accelerated electrochemical reaction kinetics and better rate capability (Fig. 6c–e) due to the cooperative work of the nitrogen active sites and conductive Ni network. The rate performance of both electrodes (S mass loading: 2.5 mg cm^−2^) at different current rates from 0.1 to 2 C is illustrated in Fig. 6c. The Ni@N-CNSs/S electrode delivers higher discharge capacities (1350, 1133, 1045, 930, and 816 mAh g^−1^ at 0.1, 0.2, 0.5, 1, and 2 C) than the N-CNSs/S counterpart (1250, 987, 856, 769, and 690 mAh g^−1^ at 0.1, 0.2, 0.5, 1, and 2 C). Obviously, the Ni@N-CNSs/S electrode exhibits obvious high-rate improvement, owing to its better conductive network and enhanced physisorption and chemisorption ability to the soluble polysulfide species. Both discharging curves of N-CNSs/S and Ni@N-CNSs/S electrodes (0.1 C) show two charge/discharge plateaus (Fig. 6d), which is consistent with the CV results above. The Ni@N-CNSs/S electrode shows higher discharge plateau voltage and lower charge plateau voltage, as compared with N-CNSs/S electrode, implying its smaller voltage drop and lower polarization. Moreover, the Ni@N-CNSs/S electrode shows superior cycling stability with an initial discharging capacity of 1350 mAh g^−1^ with a capacity retention of ~ 87% at 0.1 C after 200 cycles (Fig. 6e), while the corresponding value of the N-CNSs/S electrode is 1250 mAh g^−1^ with a capacity retention of 76%. The obtained values of Ni@N-CNSs/S electrode are also much better than that of other carbon/S powder electrodes (e.g., hollow carbon nanospheres/S [37], carbon spheres/S [39], S/C nanospheres [36, 40]) (Table S1). As for the Coulombic Efficiency (CE) analysis, after 200 cycles, the CE value of Ni@N-CNSs/S electrode maintains 98.5%, higher than that of N-CNSs/S (97.8%). After 200 cycles at 0.1 C, the whole composite structure is basically well preserved (Fig. S4). In addition, after 500 cycles at 1 C, the Ni@N-CNSs/S electrode shows a capacity of ~ 699 mAh g^−1^, higher than that of the N-CNSs/S counterpart (477 mAh g^−1^), indicating its good high-rate stability (Fig. S5). When the loading mass of sulfur is increased up to 4.5 mg cm^−2^, the Ni@N-CNSs/S electrode still exhibits good cycling life with a capacity of 839 mAh g^−1^ at 0.1 C after 500 cycles (Fig. S6), better than that of the N-CNSs/S counterpart (640 mAh g^−1^ at 0.1 C after 500 cycles).

The enhanced performance is also verified by morphology comparison of lithium metal anode after cycling at 0.1 C for 100 cycles. As shown in Fig. S7a, c, the Li anode cycled with Ni@N-CNSs/S cathode for 100 cycles shows lower roughness than the counterpart coupled with N-CNSs/S cathode, verifying the effective suppression of the polysulfides due to the synergistic effect between N-doped CNSs architecture and polar superficial NiO layer on Ni. By comparing the color of separators after cycles, the cycled separator with Ni@N-CNSs/S cathode exhibits a color of lighter yellow (inset in Fig. S7a, c). Furthermore, according to the EDS mapping images of S (Fig. S7b, d), less polysulfides have diffused to the Li anode coupled with Ni@N-CNSs/S cathode, indicating its better suppressing effect on the soluble polysulfides.

**Fig. 7:**
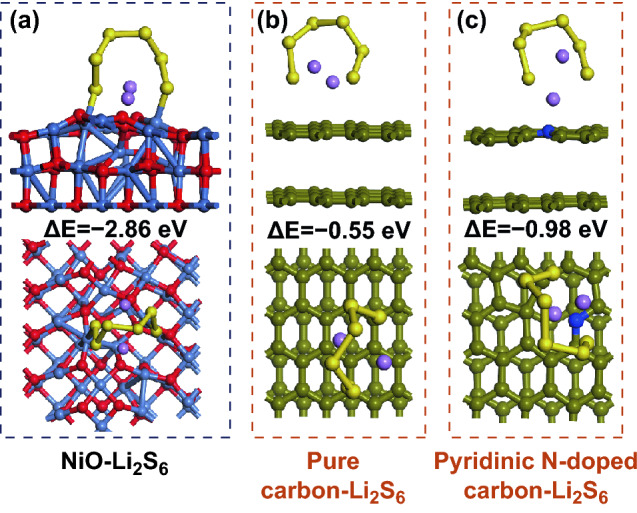
DFT calculations on adsorption energies between NiO, pure carbon, and pyridinic-N-doped carbon with Li_2_S_6_

The outstanding electrochemical performance of Ni@N-CNSs/S electrode is mainly owing to the following positive factors: (1) Binder-free conductive characteristics. The continuous conductive network of Ni@N-CNSs without additives and binders can effectively decrease inner charge transfer resistance by avoiding undesirable interfaces, and thereby enhance the electrical conductivity of the electrode and reaction kinetics [41–43]. (2) Physical adsorption and block to the soluble lithium polysulfides. Cross-linked multilayer carbon nanospheres films provide a physical “dam” to entrap the soluble polysulfide species and decreases the irreversible capacity during the charge/discharge processes. (3) Chemical adsorption to the polysulfides. Doped nitrogen heteroatoms and polar superficial NiO on Ni cannot only enhance the electronic conductivity of the matrix, but also synergistically exhibit strong chemical interaction with the long-chain polysulfides, resulting in higher utilization of active materials and stable cycling life during the redox reaction processes. In our case, the function of Ni shell should be highlighted as follows. On one hand, the Ni layer on N-CNSs can establish good electron transfer path from the bottom to the top forming omnibearing conductive network. According to four-point probe method, the electrical conductivity of Ni@N-CNSs is 8.7 × 10^4^ S m^−1^, much higher than that of N-CNSs films (0.9 × 10^3^ S m^−1^). On the other hand, the superficial NiO at the Ni layer has strong adsorption ability to the soluble polysulfides to maintain good electrochemical performance. Figure 7 shows the adsorption energetics of the optimized adsorption configurations with Li_2_S_6_ molecular on thin NiO layer (NiO is the oxidized layer on the surface of Ni), pure carbon and N-doped carbon. The adsorption energy of Li_2_S_6_ on NiO is about − 2.86 eV, higher than the values obtained from N-doped carbon (− 0.98 eV) and pure carbon (− 0.55 eV) as well as the adsorption energy value between 1,2-dimethoxyethane (DME)-Li_2_S_6_ (− 0.77 eV) [44]. It suggests that the presence of N doping and superficial NiO on the Ni layer coating can greatly enhance the chemical affinity to the soluble Li_2_S_6_, leading to boosted rate performance and long-term cycles.

High-resolution S 2*p* spectra of pristine Li_2_S_6_ and Ni@N-CNSs-Li_2_S_6_ are shown in Fig. S8. The pristine Li_2_S_6_ exhibits two typical sulfur environments at 161.6 and 163.1 eV owing to terminal (S_T_^−1^) and bridging (S_B_^0^) sulfur atoms [45], respectively. In contrast, after coupling with Ni@N-CNSs host, the peak of S_T_^−1^ positively shifts to higher binding energy of 162.9 eV, suggesting a decrease in electron density on S_T_^−1^ in Ni@N-CNSs-Li_2_S_6_. This result indicates that Ni@N-CNSs host has strong chemical adsorption to S_T_^−1^. An additional S_B_^0^-C species is detected at 164.4 eV, due to the disproportionation of Li_2_S_6_ into S^0^. Furthermore, the peaks (168.2 eV) of polythionate and S-complex species appear in Ni@N-CNSs-Li_2_S_6_ due to intermediate redox reactions [45]. Given all that, it is justified that Ni@N-CNSs host can effectively entrap polysulfides to maintain good electrochemical performance. A simple adsorption test was conducted for Ni@/N-CNSs and N-CNSs (Fig. S9). After 18 h, the polysulfide solution with Ni@N-CNSs becomes transparent, while the counterpart still shows light yellow. It is indicated that Ni@/N-CNSs host exhibits much better adsorption ability to the soluble polysulfides.

## Conclusion

In summary, we have proposed a new hydrothermal-atomic layer deposition method to synthesize binder-free Ni@N-CNSs films as sulfur host for lithium–sulfur batteries. The binder-free multilayer N-CNSs films not only perform enhanced electronic conductivity, but also provide physical block toward the soluble long-chain polysulfides. With the deposition of ALD-Ni shell, the synergistic work of nitrogen active sites and polar Ni adsorbents further increases the intrinsic reactivity kinetics during the redox reactions and offers a strong chemical adsorption to the polysulfide intermediates. After rational combination with sulfur, the as-synthesized Ni@N-CNSs/S cathode shows enhanced overall electrochemical performance with higher high-rate capacity and better cycling life due to the well-designed hybrid host. Our rational design of binder-free hybrid sulfur host may break the new ground in terms of high-performance cathode for lithium–sulfur batteries.

## Electronic supplementary material

Below is the link to the electronic supplementary material.

Supplementary material 1 (PDF 587 kb)
