# Supplementary material for: Atomic Layer Deposition-Assisted Construction of Binder-Free Ni@N-Doped Carbon Nanospheres Films as Advanced Host for Sulfur Cathode
Source: Nanomicro Lett. 2019 Aug 2;11:64. doi: 10.1007/s40820-019-0295-8 (PMC7770870; doi:10.1007/s40820-019-0295-8)
Supplement: Supplementary file 1 — Supplementary material 1 (PDF 587 kb) [file 40820_2019_295_MOESM1_ESM.pdf]

Supplementary Information for

# Atomic Layer Deposition Assisted Construction of Binder-Free Ni@N-Doped Carbon Nanospheres Films as Advanced Host for Sulfur Cathode

Jun Liu[1, *], Aixiang Wei[1, 2], Guoxiang Pan[3], Qinqin Xiong[4], Fang Chen[5], Shenghui Shen[6], Xinhui Xia[6, 7, *]

[1]Guangdong Provincial Key Laboratory of Functional Soft Condensed Matter, School of Materials and Energy, Guangdong University of Technology, Guangzhou 510006, People's Republic of China

[2]Department of Information Science, Xinhua College of Sun Yat-sen University, Guangzhou 510520, People's Republic of China

[3]Department of Materials Chemistry, Huzhou University, Huzhou 313000, People's Republic of China

[4]College of Materials and Environmental Engineering, Hangzhou Dianzi University, Hangzhou 310018, People's Republic of China

[5]Department of Chemistry, Zhejiang University, Hangzhou 310027, People's Republic of China

[6]State Key Laboratory of Silicon Materials, Key Laboratory of Advanced Materials and Applications for Batteries of Zhejiang Province, and Department of Materials Science and Engineering, Zhejiang University, Hangzhou 310027, People's Republic of China

[7]Key Laboratory of Advanced Energy Materials Chemistry (Ministry of Education), College of Chemistry, Nankai University, Tianjin 300071, People's Republic of China

*Corresponding authors. E-mail: gdutliu@gdut.edu.cn (Jun Liu); helloxxh@zju.edu.cn (Xinhui Xia)

## Supplementary Figures and Table

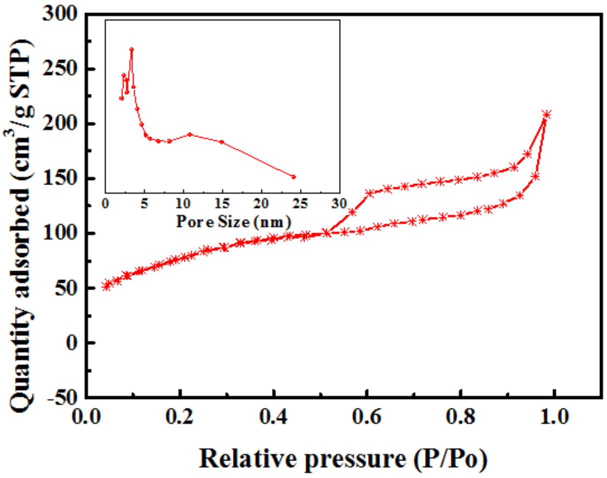


**Fig. S1** Adsorption-desorption isothermal curves of N-CNSs films (pore size distribution in inset)

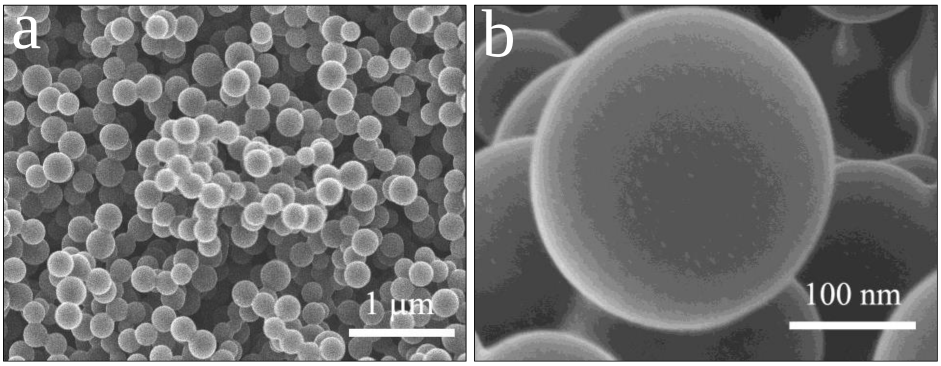


**Fig. S2 a, b** SEM images N-CNSs/S films

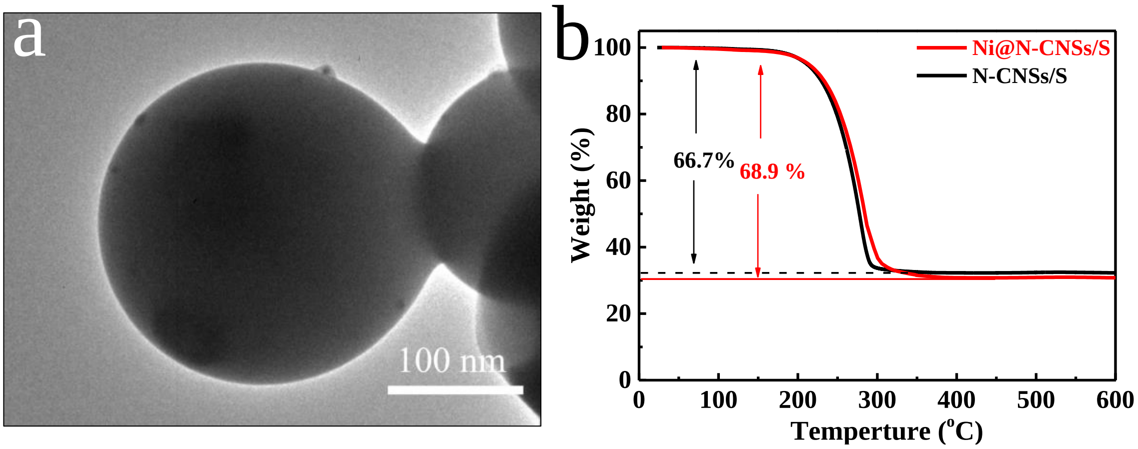


**Fig. S3 a** TEM images N-CNSs/S. **b** TGA curves of Ni@N-CNSs/S and N-CNSs/S electrodes

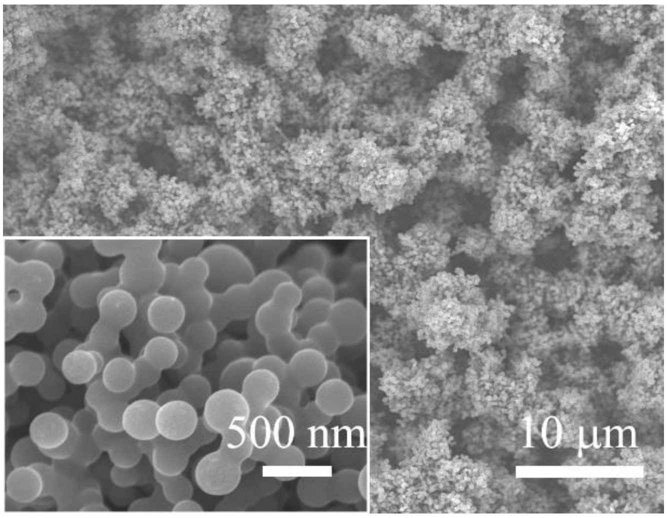


**Fig. S4** SEM image of Ni@N-CNSs/S electrode after 200 cycles at 0.1 C

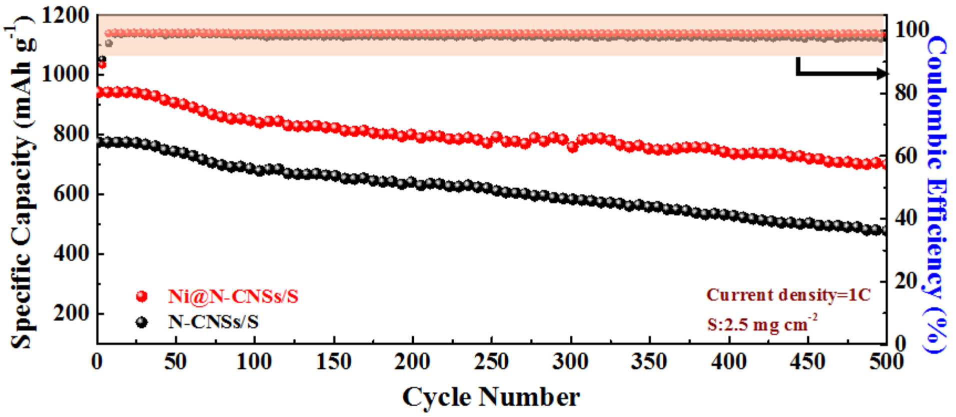


**Fig. S5** Cycling performance and coulombic efficiency of N-CNSs/S and Ni@N-CNSs/S electrodes with S mass of 2.5 mg cm$^{-2}$ at 1 C

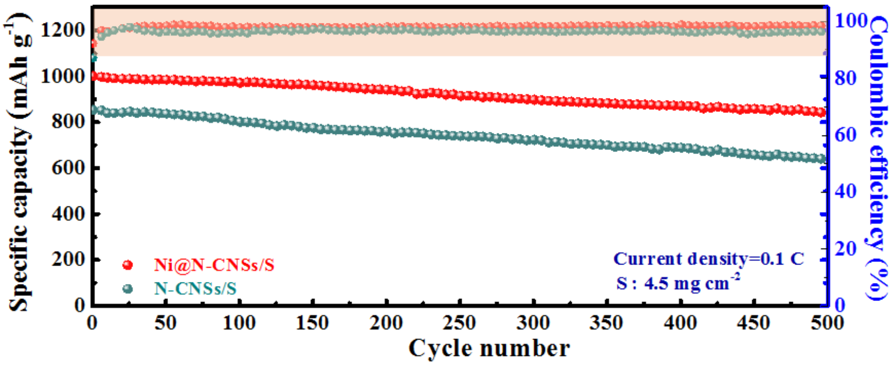


**Fig. S6** Cycling performance and coulombic efficiency of N-CNSs/S and Ni@N-CNSs/S electrodes with S mass of 4.5 mg cm$^{-2}$ at 0.1 C

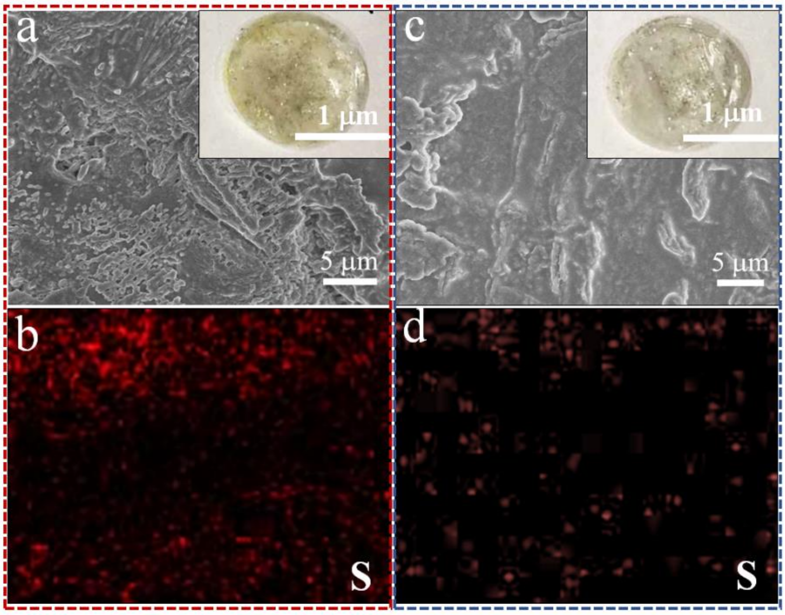


**Fig. S7** SEM image and mapping image of S for cycled lithium anodes after 100 cycles at 0.1 C: **a, b** Cycled lithium anode with N-CNSs/S; **c, d** Cycled lithium anode with Ni@N-CNSs/S cathode (digital photos of cycled separators in inset)

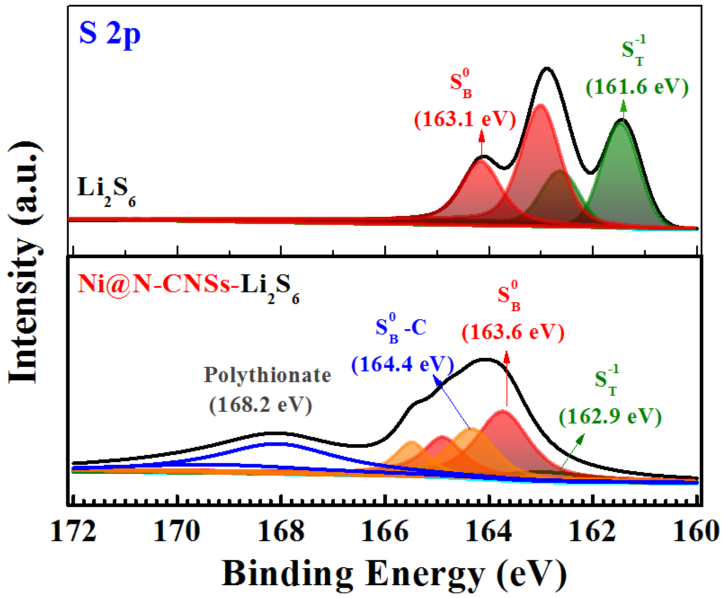


**Fig. S8** XPS analysis of pristine $Li_2S_6$ and Ni@N-CNSs-$Li_2S_6$ samples: S 2p spectra

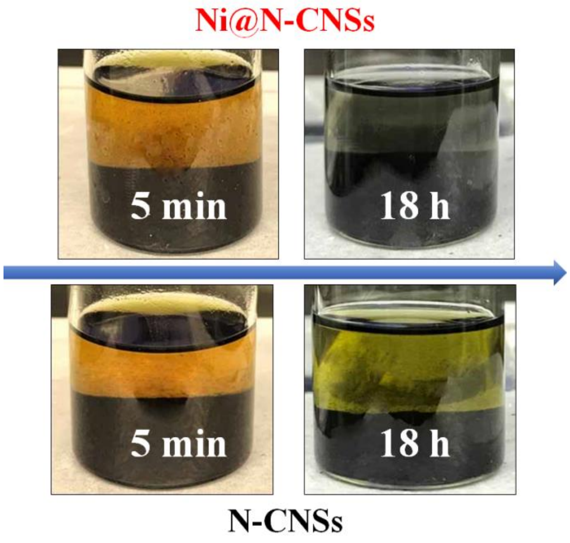


**Fig. S9** Adsorption test for $Li_2S_6$ solution with Ni@/N-CNSs and N-CNSs

**Table S1** Electrochemical performance of other carbon/S composite cathodes

| Samples | Initial capacity | Cyclability (mAh $g^{-1}$) |
|---|---|---|
| CNSs/S powder [S1] | 1031 mAh $g^{-1}$ at 0.5 C | 477 mAh $g^{-1}$ after 100 cycles at 0.5 C |
| Porous CNSs/S powder [S2] | 1150 mAh $g^{-1}$ at 1 C | 680 mAh $g^{-1}$ after 100 cycles at 1 C |
| CNTs/graphene/S powder [S3] | 829 mAh $g^{-1}$ at 0.5 C | 89% retention after 200 cycles at 0.5 C |
| S/C nanosphere (PSCs) powder [S4] | 809 mAh $g^{-1}$ at 0.5 C | 775 mAh $g^{-1}$ after 200 cycles at 0.5 C |
| Hollow CNSs/S powder [S5] | ~1318 mA h $g^{-1}$ at 0.5 C | ~700 mAh $g^{-1}$ after 200 cycles at 0.5 C |
| TiO@CNSs/S powder [S6] | 1285 mAh $g^{-1}$ at 0.1 C | 76% retention after 200 cycles at 0.1 C |
| Yolk–shell CNSs/S powder [S7] | 1106 mAh $g^{-1}$ at 0.05 C | 832 mAh $g^{-1}$ after 200 cycles at 0.05 C |
| CNSs-trithiocyanuric acid /S powder [S8] | 1227 mAh $g^{-1}$ at 0.2 C | 443 mAh $g^{-1}$ after 200 cycles at 0.2 C |
| S@C@$MnO_2$/S powder [S9] | 1345 mAh $g^{-1}$ at 0.1 C | 1043 mAh $g^{-1}$ after 200 cycles at 0.1 C |
| N-CNSs@$MnO_2$/S powder [S10] | 1249 mAh $g^{-1}$ at 0.5 C | 1110 mAh $g^{-1}$ after 200 cycles at 0.1 C |
| **This work** | **1350 mAh $g^{-1}$ at 0.1C** | **1175 mAh $g^{-1}$ after 200 cycles at 0.1 C** |

## Supplementary References

[S1]Y. Qu, Z. Zhang, X. Wang, Y. Lai, Y. Liu, J. Li, A simple SDS-assisted self-assembly method for the synthesis of hollow carbon nanospheres to encapsulate sulfur for advanced lithium–sulfur batteries. J. Mater. Chem. A **1**, 14306-14310 (2013). https://doi.org/10.1039/c3ta13306k

[S2]G. He, S. Evers, X. Liang, M. Cuisinier, A. Garsuch, L.F. Nazar, Tailoring porosity in carbon nanospheres for lithium–sulfur battery cathodes. ACS Nano **7**, 10920-10930 (2013). https://doi.org/10.1021/nn404439r

[S3]L. Zhu, H.-J. Peng, J. Liang, J.-Q. Huang, C.-M. Chen et al., Interconnected carbon nanotube/graphene nanosphere scaffolds as free-standing paper electrode for high-rate and ultra-stable lithium–sulfur batteries. Nano Energy **11**, 746-755 (2015). https://doi.org/10.1016/j.nanoen.2014.11.062

[S4]H. Hu, H. Cheng, Z. Liu, G. Li, Q. Zhu, Y. Yu, In situ polymerized PAN-assisted S/C nanosphere with enhanced high-power performance as cathode for lithium/sulfur batteries. Nano Lett. **15**, 5116-5123 (2015). https://doi.org/10.1021/acs.nanolett.5b01294

[S5]H.H. Nersisyan, S.H. Joo, B.U. Yoo, D.Y. Kim, T.H. Lee et al., Combustion-mediated synthesis of hollow carbon nanospheres for high-performance cathode material in lithium-sulfur battery. Carbon **103**, 255-262 (2016). https://doi.org/10.1016/j.carbon.2016.03.022

[S6]Z. Li, J. Zhang, B. Guan, D. Wang, L.M. Liu, X.W. Lou, A sulfur host based on titanium monoxide@carbon hollow spheres for advanced lithium–sulfur batteries. Nat. Commun. **7**, 13065 (2016). https://doi.org/10.1038/ncomms13065

[S7]T. Yang, Y. Zhong, J. Liang, M. Rahman, W. Lei, Y. Chen, M. Monteiro, Z. Shao, J. Liu, Hierarchical porous yolk–shell carbon nanosphere for high-performance lithium–sulfur batteries. Part. Part. Syst. Char. **34**, 1600281 (2017). https://doi.org/10.1002/ppsc.201600281

[S8]S.-Z. Zeng, Y. Yao, X. Zeng, Q. He, X. Zheng, S. Chen, W. Tu, J. Zou, A composite of hollow carbon nanospheres and sulfur-rich polymers for lithium-sulfur batteries. J. Power Sources **357**, 11-18 (2017). https://doi.org/10.1016/j.jpowsour.2017.04.092

[S9] L. Ni, G. Zhao, G. Yang, G. Niu, M. Chen, G. Diao, Dual core-shell-structured S@C@$MnO_2$ nanocomposite for highly stable lithium-sulfur batteries. ACS Appl. Mater. Interfaces **9**, 34793-34803 (2017). https://doi.org/10.1021/acsami.7b07996

[S10] M. Chen, Q. Lu, S. Jiang, C. Huang, X. Wang, B. Wu, K. Xiang, Y. Wu, $MnO_2$ nanosheets grown on the internal/external surface of N-doped hollow porous carbon nanospheres as the sulfur host of advanced lithium-sulfur batteries. Chem. Eng. J. **335**, 831-842 (2018). https://doi.org/10.1016/j.cej.2017.11.039
